# Fundus autofluorescence in premature infants

**DOI:** 10.1038/s41598-021-88262-z

**Published:** 2021-04-23

**Authors:** Guillermo Salcedo-Villanueva, Yurico Lopez-Contreras, Ana Gonzalez-H. Leon, Juan C. Romo-Aguas, Gerardo Garcia-Aguirre, Linda A. Cernichiaro-Espinosa, Maria A. Martinez-Castellanos, Hugo Quiroz-Mercado

**Affiliations:** 1 Retina Department, Asociación Para Evitar La Ceguera en México, IAP, Vicente García Torres #46, Col. San Lucas, CP 04030 Mexico City, Mexico; 2 School of Medicine and Health Sciences, Tecnologico De Monterrey, Mexico City, Mexico

**Keywords:** Physiology, Visual system, Retina

## Abstract

To describe fundus autofluorescence (FAF) patterns in premature infants and to determine whether FAF increases gradually with increasing post-gestational age. This was a cross-sectional, observational and descriptive case series. FAF images were obtained from patients screened for Retinopathy of Prematurity. The presence of the following hypo-autofluorescence areas/structures was graded and ranked: macular pigment (foveal centre), optic nerve head, peripapillary vessels/vascular arcade (PP/VA), and equatorial vessels (EqV). Ranks were attributed to the number of structures visualized from the posterior pole towards the periphery. The rank of FAF could then be analysed by Spearman’s correlation against age. Additionally, patients were divided by age into group 1 (< 40 weeks of corrected gestational age (WCGA)) and group 2 (> 40 WCGA). Differences between groups were tested with the Mann–Whitney U test. Thirteen patients were analysed. The mean WCGA at examination was 47.85 weeks. Spearman’s correlation showed a strong positive correlation (r = 0.714) (P = 0.006) of FAF and WCGA. The Mann–Whitney U test revealed that the PP/VA and EqV were significantly more visible at > 40 WCGA than at < 40 WCGA (8.0 [P = 0.016] and 7.5 [P = 0.03], respectively). Patterns of FAF are described for the first time in premature infants. FAF increases gradually with age and centrifugally from the posterior pole towards the equator in premature infants.

## Introduction

Fundus autofluorescence (FAF) is the inherent emission of visible light that occurs when lipofuscin found in the retinal pigment epithelium (RPE) is excited between 400 and 590 nm and then emits light at longer wavelengths (520–800 nm)^1–3^. Lipofuscin thus acts as a mixture of fluorescent molecules.

Lipofuscin is the main fundus fluorophore^1,4^. It consists of a mixture of several bisretinoid fluorophores that form due to an unwanted reaction of retinaldehyde (vitamin A aldehyde). The bisretinoids that have been described so far have similar structures but vary in terms of excitation spectra^5–8^. Due to its distinctive molecular structure, lipofuscin cannot be entirely degraded, leading to its accumulation inside RPE cells. Fluorophore concentrations increase with age in a healthy individual^4,9^, while several diseases of the photoreceptors or RPE may alter the amount of bisretinoids formed. This makes FAF an important physiological and diagnostic tool^10,11^. FAF patterns in the normal adult population have been widely described^12–14^.

The lipofuscin present in the lysosomal compartment of the RPE in part reflects the accumulation of these fluorophores throughout life, since they appear to be refractory to enzyme digestion^3^. Hence, it would be expected that the FAF intensity (reflecting the amount of fluorophores in the RPE) should increase from the moment of birth onwards. However, to the best of our knowledge, no previous report has studied FAF patterns in infants, which could demonstrate how the gradual accumulation of fluorophores increases fluorescence with advancing age during the first year of life. Hence, the purpose of this study was to describe FAF patterns in an infant population and to determine whether FAF increases gradually with increasing post-gestational age.

## Methods

This was a cross-sectional, observational and descriptive case series. We included infants who were referred to our Pediatric Retina Department for routine ROP/postnatal screening. The study was carried out in accordance with Good Clinical Practice guidelines with strict adherence to the principles of the Declaration of Helsinki. Institutional Review Board (IRB) approval was obtained for this study (CONBIOÉTICA-09-CEI-005–20,170,306). Prior to examination and image acquisition, the parents of the screened infants signed informed consent forms to participate in the study and to publish related data and/or images.

Our hospital works as a non-profit, privately funded ophthalmological referral centre in Mexico City, where systemically stable premature infants are referred for ROP/postnatal screening. Examination is performed under pupillary dilation by indirect ophthalmoscopy, and when applicable, fundus images are obtained. As per the internal protocol of the Pediatric Retina Department, from January to October 2019, all infants were imaged with the Optos Daytona system (Optos plc. Dunfermline, Scotland), obtaining ultra-wide-field images (UWFIs). To obtain FAF, the Daytona System uses a λ532 nm confocal scanning laser ophthalmoscope (cSLO) for excitation and captures fundus emissions in the range of λ570 nm to λ780 nm^15^. Vital signs were monitored throughout the examination and image acquisition by pulse oximetry. If the patient showed signs of haemodynamic decompensation such as decreased oxygen saturation, an increased heart rate (> 200 bpm) or any other condition where the neonatologist contraindicated it, images were not taken.

Patients were included if they had clear optical media and no ocular diseases except for ROP (mild ROP, type 1 ROP or type 2 ROP). Patients with a known family history of a hereditary retinal disease, the presence of ROP with Plus disease and/or the need for treatment during their follow-up were excluded. Images were eliminated from the study if no view of the fundus was obtained due to motion artefacts. Final image analysis was therefore performed only in patients who were kept under observation, with no intervention.

### Acquisition of FAF in infants using UWFIs

Briefly, to acquire images in premature infants, the protective lid from the Optos Daytona system must be removed. A topical mydriatic agent (tropicamide (50 mg/ml) with phenylephrine (8 mg/ml)), as well as topical anaesthesia (tetracaine (5 mg/ml)), was administered with prior assurance of adequate pupillary reflexes and light response. Patients were monitored using a pulse oximeter at all times. The infant was placed (tucked) inside a blanket to secure the upper and lower extremities; he or she was then set in an upright position in front of the system’s aperture that leads to the cSLO mirror. A lid speculum was used to open the eyelids. Normally, three people were involved in this process: one to hold the infant’s body, a second to hold the infant’s head in place and move it accordingly near the system’s acquisition aperture, and a third to manually take the UWFIs. Image acquisition took approximately 0.4 s, allowing a brief but sufficient window to obtain pseudocolour images or FAF, even if the infant was moving.

### FAF grading

Based on the absence of published literature on FAF patterns in premature infants and considering that FAF should increase gradually with age, we decided to observe the presence or absence of fluorescence in the different structures of the posterior pole. Normally, when visualizing an FAF image, there is near absolute contrast between the structures emitting light (mainly the RPE) and the structures or areas blocking fluorescence (i.e., macular pigment, retinal vessels, and optic nerve head [ONH]). We hypothesized that FAF should, at some point, be completely absent in the prenatal development of a normal RPE before light activation of the visual cycle and gradually increase as POS undergoes phagocytosis and lipofuscin begins to accumulate. Therefore, some areas of the fundus would begin to show more fluorescence than others. With this in mind, we considered that the area with increased metabolic activity would be the sub-macular RPE and that the fluorescence would increase centrifugally.

Finally, if we consider that the formation of the fluorophores responsible for FAF begins when visual cycle functioning allows the production of 11-cis-chromophore, fluorescence emission would be very low at this point. We decided that the structures to be examined would be the ones blocking fluorescence, which would contrast the emission of light from the RPE (i.e., macular pigments would block the light from the subfoveal RPE and allow the macula to be identified; the ONH would contrast with the fluorescence from its surrounding peripapillary retina; the vascular arcades would contrast the fluorescence from within the macula and from the extra-macular retina; and the equatorial retina would be identified if the retinal vessels blocked the fluorescence from the RPE in that region). The identification of these areas/structures would yield qualitative data that we could eventually use to rank and measure FAF (as explained below).

Images were thus graded dichotomously into “yes” or “no” according to the following patterns of structures observed and the presence of hyper-autofluorescence (hyper-AF) or hypo-autofluorescence (hypo-AF):

1. In general, was FAF observed?
2. Was optic nerve head hypo-AF observed (peripapillary hyper-AF)?
3. Was macular pigment hypo-AF observed (macular hyper-AF)?
4. Was peripapillary vessels/vascular arcade (PP/VA) hypo-AF observed (macular and para-macular hyper-AF)?
5. Was equatorial retinal vessels (EqV) hypo-AF observed (equatorial RPE hyper-AF)?

### Image and statistical analyses

Images were graded by consensus from two retina specialists (GSV and MAMC) who were blinded to the patient’s background information (especially age). Based on this initial grading, ranks were attributed to the number of structures visualized from the posterior pole towards the periphery. We chose to use ordinal variables since hyper-AF would not be expected at the equator without observing it first in the posterior pole. Hence, three ranks were created: rank (1) visualization of the ONH exclusively; rank (2) visualization of the ONH and PP/VA; and rank (3) visualization of the ONH + PP/VA and EqV. The ranks could then be analysed by Spearman’s correlation to determine whether a positive correlation exists with increasing age and augmenting fluorescence due to the accumulation of lipofuscin.

Finally, from the whole set of images, two groups were formed to divide and compare differences between weeks of corrected gestational age (WCGA) in terms of the visualization of structures by FAF: group 1 (< 40 WCGA) and group 2 (> 40 WCGA). Mann–Whitney U tests were performed to determine differences between groups.

From the small data set and assuming non-normal distribution, as well as analysis by ranks and ordinal variables, non-parametric tests were performed: Spearman’s correlation and Mann–Whitney U tests. Data were recorded into spreadsheets. Statistical analysis was performed with SPSS (Ver. 22. IBM Corp., Armonk, USA).

### Ethics approval

This research project was approved by the Institutional Review Board of the Asociación Para Evitar la Ceguera en México (CONBIOÉTICA-09-CEI-005-20170306). The study was performed in accordance with the ethical standards laid down in the 1964 Declaration of Helsinki and its later amendments.

### Consent to participate

Written informed consent was obtained from all parents or legal guardians.

### Consent to Publish

Patients signed informed consent regarding publishing their data.

## Results

We analysed 13 images from 13 patients who met the inclusion and exclusion criteria (9 male and 4 female patients who were screened for ROP, with a mean gestational age (GA) at birth of 29.54 weeks) (median: 29.5 weeks GA; standard deviation [SD]: 2.9; range: 25.5–34). The mean weight at birth was 1,194.08 g (median: 1150 g; SD: 263.14; range: 860–1730). The mean WCGA at the time of examination was 47.85 weeks (median: 51; SD: 9.01; range: 36–62). Diagnoses at the initial examination were as follows: 4 patients with zone II/stage 1 ROP; 1 patient with zone II/stage 2 ROP; 4 patients with zone III/stage 1 ROP; and 4 patients without ROP. No patient had Plus disease, and no patient required treatment during their follow-up.

**Figure 1:**
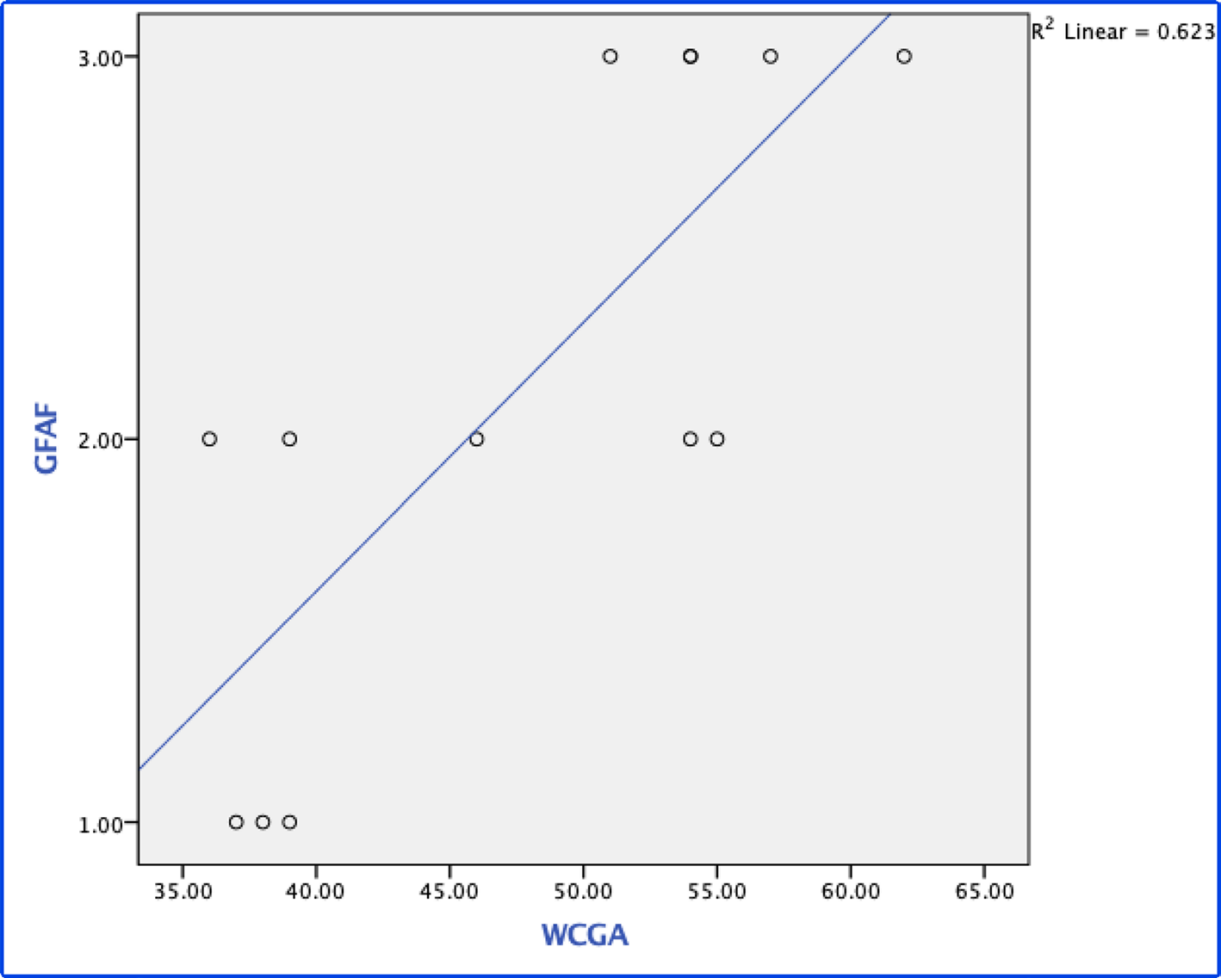
Scatter plot of fundus autofluorescence rank vs. weeks of corrected gestational age. Rank 1: On the optic nerve head (ONH) was visible; rank 2: only the ONH and peripapillary vessels/vascular arcade (PP/VA) were visible; and rank 3: the ONH + PP/VA and equatorial vessels (EqV) were visible. Spearman’s correlation: r = 0.714 (*P* = 0.006).

The identification of hyper/hypo-AF structures is shown in Table 1. Only 5 patients had visible hypo-AF macular pigment (38.4%); all 13 patients had an identifiable hypo-AF ONH (100%); ten patients had a hypo-AF PP/VA (76.92%); and 5 patients had hypo-AF EqV (38.46%). Only formed structures (ONH and retinal vasculature) were ranked into grades of FAF based on our hypothesis that fluorophores should accumulate in a centrifugal pattern: rank (1) only the ONH was visible; rank (2) the ONH + PP/VA were visible; and rank (3) the ONH + PP/VA + EqV were visible (macular pigment was not included in this analysis, as discussed below). Spearman’s rank correlation was performed on ranks of FAF against WCGA, resulting in a strong, positive correlation (r = 0.714) (*P* = 0.006), indicating that with more WCGA, there is more fluorescence, which helps identify hypo-AF structures. The linear correlation is shown in a scatter plot in Fig. 1.

**Table 1:** Identification of hypo-AF structures.

Case number	Gender	Weight at birth in grams	WCGA	ONH Hypo-AF	Macular Pigment Hypo-AF	PP/VA Hypo-AF	EqV Hypo-AF
1	Male	1280	36	Yes	No	Yes	No
2	Male	1020	37	Yes	No	No	No
3	Male	1540	38	Yes	No	No	No
4	Male	1460	39	Yes	Yes	Yes	No
5	Male	950	39	Yes	No	No	No
6	Male	1238	46	Yes	Yes	Yes	No
7	Female	860	51	Yes	No	Yes	Yes
8	Male	1040	54	Yes	Yes	Yes	Yes
9	Female	1000	54	Yes	No	Yes	No
10	Male	940	54	Yes	No	Yes	Yes
11	Female	1150	55	Yes	Yes	Yes	No
12	Male	1315	57	Yes	No	Yes	Yes
13	Female	1730	62	Yes	No	Yes	Yes
		M:11,294.08 g SD: 263.14	M: 47.85 SD: 9.01	Yes: 100%	Yes: 38.4%	Yes: 76.92%	Yes: 38.46%

*Hypo-AF* hypo-autofluorescence, *WCGA* weeks of corrected gestational age, *ONH* optic nerve head, *PP/VA* peripapillary/vascular arcades, *EqV* equatorial vessels, *M* mean, *SD* standard deviation.

To better understand the influence of age (expressed in WCGA) on FAF, we divided our dichotomous variables into two groups: group 1 (< 40 WCGA) and group 2 (> 40 WCGA) (Table 2). Mann–Whitney U (MW-U) tests were performed to determine the influence of age on the presence of FAF (again, determined by visualization (or not) of the structures). Macular pigment resulted in a non-significant MW-U test value of 16.5 (*P* = 0.523); therefore, there was no difference in the presence of macular pigment between the < 40 WCGA and > 40 WCGA groups. The PP/VA resulted in a significant MW-U test value of 8.0 (*P* = 0.016). EqV resulted in a significant MW-U test value of 7.5 (*P* = 0.03). Therefore, the PP/VA and EqV were significantly more visible at > 40 WCGA than at < 40 WCGA.

**Table 2:** Comparison of visualized structures < 40 WCGA vs. > 40 WVGA.

Visualized Structures	Groups	Mann–Whitney U test
Macular Pigment	< 40 WCGA: 1 case	16.5 (*P* = 0.523)
	> 40 WCGA: 3 cases	
PP/VA	< 40 WCGA: 2 cases	8.0 (P = 0.016*)
	> 40 WCGA: 8 cases	
EqV	< 40 WCGA: 0 cases	7.5 (P = 0.03*)
	> 40 WCGA: 5 cases	

*WCGA* weeks of corrected gestational age, *PP/VA* peripapillary/vascular arcades, *EqV* equatorial vessels. *Statistical significance*, *P* < 0.05.

**Figure 2:**
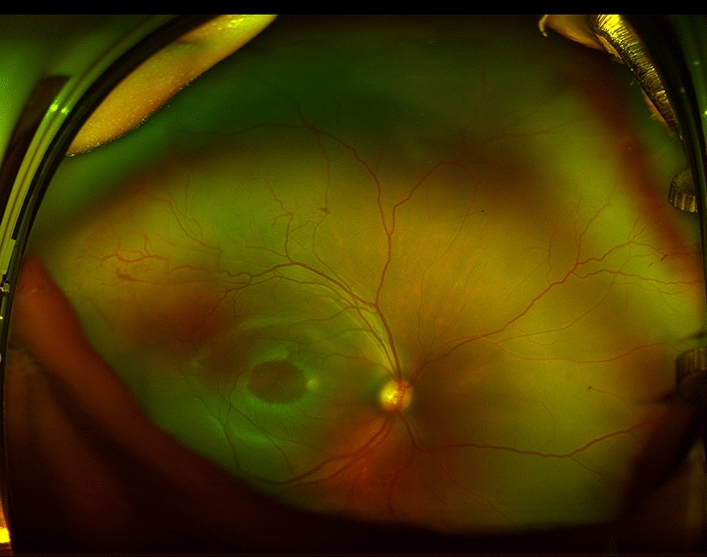
Ultra-wide-field pseudocolour image of a male patient (case #4) weighing 1460 g at birth (32 weeks of gestational age at birth and 39 weeks of corrected gestational age at the first examination) who was diagnosed with zone II/stage 1 ROP. The image shows angiogenesis towards the temporal mid-peripheral retina. Regardless of the lack of visualization of the far periphery, details of the posterior pole’s development and maturation, such as complete vascularization of the temporal arcades and foveal depression, can be observed.

**Figure 3:**
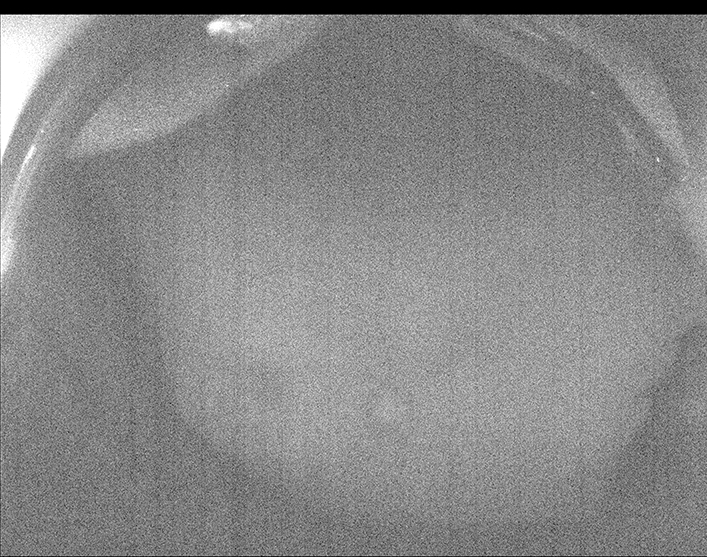
Fundus autofluorescence of case #4 (39 weeks of corrected gestational age), where low-grade hyper-autofluorescence contrasts with the hypo-autofluorescent structures that block the emission of light: optic nerve head, macular pigment, peripapillary vessels and vascular arcade. Note that vessels are not observed in the equatorial retina (approximate peak emission for fluorophores: λ610 nm; range for excitation maxima for the different fluorophores: λ430 nm – λ510 nm)^6^.

**Figure 4:**
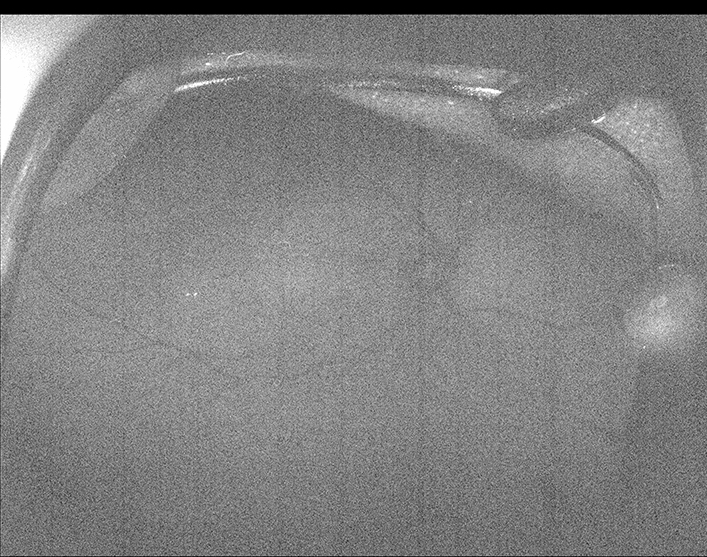
Fundus autofluorescence of case #4 at 62 weeks of corrected gestational age. Note that the retinal vessels can be followed throughout their trajectory in the temporal and nasal retina (approximate peak emission for fluorophores: λ610 nm; range for excitation maxima for the different fluorophores: λ430 nm – λ510 nm)^6^.

Finally, only one patient had FAF follow-up images. The case was a male patient weighing 1460 g at birth (32 weeks of GA at birth and 39 WCGA at the first examination) who was diagnosed with zone II/stage 1 ROP (Fig. 2) and then reexamined at 62 WCGA with adequate FAF images for follow-up (although his follow-up data were not included in the analysis to avoid biased results). Baseline FAF images showed mild generalized fluorescence, with hypo-AF surrounding the ONH, hypo-AF that outlined the PP/VA, and hypo-AF due to macular pigment, but the EqV were not identified (Fig. 3). At 62 WCGA, the outline of the EqV was observed and followed throughout the temporal and nasal retina (Fig. 4).

## Discussion

Based on the results from this case series of FAF in premature infants, throughout the first weeks after birth, autofluorescence seems to increase with age, from an almost absence of fluorescence emission in younger patients to the visualization of the equator and clear contrast between hyper and hypo-AF structures in older infants. The patterns of FAF observed also seem to augment centrifugally, allowing gradual identification of the anatomical landmarks that can be observed eventually with the normal accumulation of bisretinoid pigments. To the best of our knowledge, this is the first report on the patterns of FAF in premature infants.

From our results, several observations can explain how FAF seems to increase with age and centrifugally. First, in all cases, at least low-grade fluorescence was observed in the posterior pole, which contrasted with hypo-AF from the ONH, even if this fluorescence was very mild in some cases. From the whole data set, there were only 3 patients in whom the ONH was the only landmark that could be identified; all 3 patients were categorised into group 1 (< 40 WCGA) and had a mean age of 38.3 WCGA. This finding supports our hypothesis that FAF begins in the posterior pole, creating contrast against the hypo-AF ONH. On the other hand, the most peripheral structures that we graded, EqV, were not observed in any patient in group 1. The PP/VA was visualized only in 2 of 5 patients in group 1. As FAF increased with age, visualization of these other structures improved. EqV (5 patients) were observed only in group 2 (> 40 WCGA); these 5 patients presented a mean age of 55.6 WCGA (SD: 4.1), in contrast to the mean age of patients in whom only the ONH was observed. Additionally, all patients in group 2 had an identifiable PP/VA (8 patients), while only 2 patients in group 1 had an identifiable PP/VA. The comparison between groups 1 and 2 by the MW-U test revealed statistically significant differences for the PP/VA and EqV (8.0 [*P* = 0.016] and 7.5 [*P* = 0.03], respectively). Finally, Spearman’s test between age and ranks of FAF revealed a positive correlation (r = 0.714) (*P* = 0.006), indicating the presence of “higher ranks” of fluorescence as age increased.

It is important to comment on the observations of macular pigment by FAF. We initially considered macular pigment as a good hypo-AF biomarker that would help identify how the underlying hyper-AF increased. However, macular pigment is a variable AF blocker that arises from processes independent of LF, and for that reason, it would not be expected to be sufficiently consistent in its relationship with surrounding FAF. Hence, we did not use it in our classification. The macular pigments are lutein, zeaxanthin and *meso*-zeaxanthin, which are xanthophyll carotenoids that are present in photoreceptor axons and Henle’s fibre layer^16,17^. Macular pigment has been reported to be detectable in the retina in the second and third trimesters of pregnancy. However, Bernstein et al.^18^ used blue-light reflectance to analyse macular pigment in infants and children and found that macular pigment was always undetectable in the group of premature infants studied (11 cases). The cause was attributed to foveal immaturity. We believe that our results offer new knowledge on the physiology and development of the ocular fundus, especially the accumulation of fluorophores in the first weeks of life. Understanding how FAF begins in the first weeks after birth might set a precedent to investigate how macular pigment is related to age and diet, an issue that is proving to be more significant everyday as our knowledge of retina and neurodegenerative diseases also increases.

FAF has been demonstrated to be characterized by certain spectral properties^19^ and age-dependent intensification, both of which are attributed to bisretinoid pigments of the retina and RPE^8,20^. These bisretinoid pigments comprise the fluorescent constituents of lipofuscin. The pigments are formed from the reaction of 11-*cis*-retinal and all-*trans*-retinal in the POS^21^. As the photoreceptor sheds its outer segments and undergoes phagocytosis, the bisretinoids that form lipofuscin are deposited into RPE cells^3^. Lipofuscin must then begin to accumulate when the POS starts to shed, increasing with age inside the lysosomal compartment^8^.

Lipofuscin inside the RPE has been measured throughout the whole fundus, increasing from the equator to the posterior pole^9,22^, which supports our observations. In a study of 145 patients with normal retinas (and RPE) aged 15 to 80 years, Delori et al.^4^ described a positive correlation of fluorescence with age, increasing quasi-linearly up to 70 years of age, that was further confirmed with quantitative FAF^14^. Our results amplify and are consistent with these previous observations: fluorescence from lipofuscin accumulates first in the posterior pole and moves centrifugally towards the periphery. However, it is important to consider that although we aimed to observe FAF from its beginning phases in postnatal life, our cohort of patients was premature. Even if the vascularization of our patients’ retinas was in zones II and III and no patient required any subsequent treatment, our results support the fluorescence observed in developing retinas and RPE cells; however, our observations may not be applied to fully developed and mature infants.

There are very few published studies on FAF in paediatric populations, especially infants. Fahim et al.^23^ described FAF patterns in patients with achromatopsia and included a 7-month-old patient who exhibited foveal and parafoveal hyperfluorescence. Dikkaya et al.^24^ described an 18-month-old patient with RD3-related Leber congenital amaurosis. Wabbels et al.^25^ studied 50 children and teenagers aged 2–16 years with hereditary retinal diseases and used 20 healthy children aged 4–16 years as a control group. In their controls, they reported observing the expected FAF patterns of mature RPE: FAF showed a fovea that was darker than the surrounding retina and absent in the area of the ONH and large vessels. Other studies have included patients with recessive Stargardt disease (STGD1)^26^ and papilledema/pseudopapilledema^27^, but their youngest patients were 7 and 5 years old, respectively. Our case series contributes to the literature on the paediatric retina with the youngest patients observed with this method so far.

We can consider several limitations to our study, the main ones being its retrospective nature and small sample size. Data were obtained from the ROP screening protocol from our institution. Most patients did not meet the inclusion criteria, some did not have FAF images, and in the ones who did, many images had to be eliminated from the analysis due to poor image quality. Thus, there was a small number of cases to study, which could have influenced our results. Sometimes, FAF can be so subtle that it is possible that many images were not analysed due to inadequate quality. Another limitation, as stated before, is that the analysis of premature infants with mild disease could be an important confounding factor that affected our results. Only further studies in mature newborns will be able to address how normal fluorescence develops from birth onwards. Last, there could be expected differences in our findings by using different types of FAF systems with varying wavelengths of stimulating light.

In conclusion, we reported a case series of premature infants that were analysed by FAF UWFIs. Patterns of FAF were described for the first time in premature infants, demonstrating an increase in fluorescence from an almost absence of emission to clear visualization of the contrasting hyper and hypo-AF structures of the fundus that increases centrifugally from the posterior pole towards the equator.

## Data Availability

All data for this research are available at 10.17632/k5jsssjtmz.1.
